# Rare Loot Box Rewards Trigger Larger Arousal and Reward Responses, and Greater Urge to Open More Loot Boxes

**DOI:** 10.1007/s10899-019-09913-5

**Published:** 2019-11-23

**Authors:** Chanel J. Larche, Katrina Chini, Christopher Lee, Mike J. Dixon, Myra Fernandes

**Affiliations:** Department of Psychology, University of Waterloo, 200 University Avenue West, Psychology, Anthropology, and Sociology Building, Waterloo, ON N2L 3G1 Canada

**Keywords:** Gambling, Gaming, Reward reactivity, Arousal, Motivation

## Abstract

Loot boxes are a purchasable video-game feature consisting of randomly determined, in-game virtual items. Due to their chance-based nature, there is much debate as to whether they constitute a form of gambling. We sought to address this issue by examining whether players treat virtual loot box rewards in a way that parallels established reward reactivity for monetary rewards in slots play. Across two sets of experiments, we show that loot boxes containing rarer items are more valuable, arousing, rewarding and urge-inducing to players, similar to the way slots gamblers treat rare large wins in slots play. Importantly, we show in Experiment 2 that the duration of Post Reinforcement Pauses, an index of reward reactivity, are longer for boxes with rarer items. Boxes containing rarer rewards also trigger larger Skin Conductance Responses and larger force responses—indices of positive arousal. Findings of Experiment 2 also revealed that there was an increase in anticipatory arousal prior to the reveal of loot box rewards. Collectively, our results elucidate the structural similarities between loot boxes and specific gambling games. The fact that players find rarer game items hedonically rewarding and motivating has implications for potential risky or excessive loot box use for some players.

**Electronic supplementary material:**

The online version of this article (10.1007/s10899-019-09913-5) contains supplementary material, which is available to authorized users.

## Introduction

The incorporation of chance-based microtransactions (i.e., in-game purchases) in video-games has sparked concern over the potential connection between video-games and gambling. Much of this concern is centred around the incorporation of loot boxes (a form of chance-based microtransaction) into games (King and Delfabbro 2018). Loot boxes are purchasable virtual boxes comprised of randomly determined in-game virtual items that vary in value based on their rarity in the game. Recent research has established a link between problem gambling severity and expenditure related to loot boxes specifically, arguing that loot box use within games may act as a ‘gateway to gambling’ (Zendle and Cairns 2018, 2019). Although researchers contend that there are parallels between loot box purchases and gambling, little is known about how players hedonically and motivationally respond to these types of rewards at the psychological, physiological and behavioural level. Specifically, in the gambling literature, research has demonstrated that physiological arousal triggered during gameplay is the primary reinforcer of gambling behaviour, and is tightly linked to one’s urge to gamble (Brown 1986; Baudinet and Blaszczynski 2013). However, unlike traditional gambling situations, such rewards in loot boxes are non-monetary in nature. To elucidate the impact of loot box use on reward processing and motivation in players, the present research examines how avid players of a game containing loot boxes psychologically value these rewards, and further, how such rewards influence players’ arousal and hence craving (i.e., urge) to open more loot boxes.

## Structural Similarities Between Loot Boxes and Slot Machines

Researchers have often compared loot boxes with slot machines, given that they both operate on a variable-ratio reinforcement schedule, which is known to elicit a pattern of reinforced/repeated behaviours (Haw 2008). Indeed, the specific contents of any given loot box are unknown to the player, in that the items are randomly determined. A key difference between loot boxes and slots is that the items within loot boxes are valuable solely within the confines of the game. The appeal of loot boxes lies in the chance to obtain rare items that a player may wish to procure—the rarer the item the more it appears to be valued by players. The chance-determined content of loot boxes is similar to the unpredictable nature of outcomes in slot machines. In slots losses are the most common, small wins are less common, and large wins are exceedingly rare. In general, just as different slots outcomes are associated with varying monetary values that correlate with their rarity, loot boxes too contain items whose worth to the player may depend on their rarity. One of the goals of the current research will be to confirm that players do indeed find rarer items as being subjectively more valuable.

## Rewards, Arousal and Urge in the Context of Gambling

Crucially, the allure of both slots games and loot box events within video-games likely involves the different arousal signatures for these various types of outcomes. Importantly, physiological arousal (e.g., triggered by gambling wins) is associated with both the onset and maintenance of gambling behaviours and has been shown to promote the urge to gamble (Baudinet and Blaszczynski 2013). Skin conductance responses (SCRs), which measure sweat gland activity, are a well-established indicator of physiological arousal (Sharpe et al. 1995; Dixon et al. 2011; Dixon et al. 2013). Wins in slots play provoke increases in physiological arousal that are titrated to the size of the win. That is, as wins get progressively larger so to do skin conductance response magnitudes (Dixon et al. 2013). If loot boxes mimic slots outcomes, then loot boxes of varying rarity would be expected to replicate this pattern, with loot boxes containing more common (lower valued) items inducing only small amounts of arousal, and loot boxes containing rarer (higher valued) items inducing commensurately higher arousal, and hence larger SCRs.

In addition to SCRs, physiological arousal during slots play can be measured using the force one exerts on the spin button. Dixon et al. (2015) demonstrated that slots players would exert greater force on the spin button to initiate the next spin following large wins. Additionally, the force applied following wins was titrated to the win-size. Dixon et al. (2015, 2018a, b) interpreted this relation between force and win size as attributable to arousal and showed that this force measure was even more sensitive to win size than SCRs. Hence, if players had to press a mouse to continue to see more loot-box openings, we would expect that the force exerted on the mouse would be titrated to the rarity of the items in the loot box that was just viewed.

Post-reinforcement pauses (PRPs) are another means of gauging the reward value of slots outcomes (Dixon et al. 2013). PRPs are a measure of the length of time between the outcome delivery and the initiation of the next spin (Dixon et al. 2013). In slots, when players spin and lose, they tend to initiate the next spin right away. When they spin and win, they tend to pause before spinning again. As mentioned, the length of this post-reinforcement pause tends to be titrated to the size of the win—the bigger the win, the longer the pause. Players appear to pause to internally celebrate the rewarding events, which exerts a momentary inhibition of further reward-seeking behaviour (Delfabbro and Winefield 1999; Dixon et al. 2013). Therefore, we expect that loot box users would demonstrate similar PRPs in response to more valuable loot boxes.

In addition to arousal effects triggered after the outcomes are revealed, loot boxes may also trigger arousal *prior* to the outcome. Increased arousal is highly associated with anticipation of risk, but importantly also with reward (Critchley et al. 2001). In games like *Overwatch*, when a loot box is obtained, there is a brief anticipatory period in the moments leading up to the reveal of the items. Animations show the loot box shaking for a period of approximately 2 s prior to showing the items exploding out of the box. Hence, arousal might be expected to increase even before the reveal of the specific loot items. Thus, loot boxes may be particularly alluring outcomes because they may trigger a buildup of arousal prior to the outcome, followed by a further increase in arousal if the items revealed are ones coveted by the player.

Hence, both the anticipation of, and the experience of reward linked to rare events (large wins, rare loot-box items) likely play a critical role in the subjective and physiological experiences of both slot machine players and loot box users. Additionally, a number of studies have shown that different types of outcomes promote the urge to keep playing in a gambling context—a phenomenon likely mediated by this combined effect of arousal triggered before and after reward delivery. For example, in both scratch cards and slot machines, if urge to keep playing is assessed following an outcome, urge to keep playing tends to be higher following a win and lower following a loss (Stange et al. 2016, 2017a, b; Clark et al. 2012). Hence, rarer, more valuable, loots are expected to induce greater urge to open additional loot boxes versus more common and less valuable loots. As urge plays an integral role in problem gambling behaviours, demonstrating the urge-inducing properties of loot boxes would further fortify the notion of an existing relationship between loot boxes, their problematic use and gambling.

## Current Study

Overall, the current research seeks to determine whether loot box users for a particular game treat loot boxes of varying values in ways that are similar to the way slot machine gamblers treat varying sizes of wins in slots play. We chose the game *Overwatch* as our central focus as it is considered to be one of the most popular games that contains loot boxes among young adults (Guskin 2018). Across two studies, we expect participants to rate loot boxes containing rarer items as being more subjectively valuable to them. We also expect loot boxes with rarer items to be more arousing, positively valenced, rewarding, and more inducing of urge to open another box. Understanding players’ reward reactivity in response to loot boxes of varying value will aid in determining whether loot boxes elicit arousing and urge-inducing responses, which are heavily implicated in the development of problematic behaviours in gamblers. If we can show that loot box rewards are treated in much the same way that monetary outcomes are treated in slots play, it would underscore that both reward structures may lead to similar reward processing and motivational effects. In general, slots are known to lead to problematic gambling for some players, and hence are highly regulated. Therefore, showing that loot boxes are responded to similarly to slots outcomes would speak to the question of whether the loot boxes constitute a form of gambling and are in need of regulation.

## Overview of Experiment 1

To our knowledge, this is the first experiment of its kind to directly observe game player responses to loot box rewards. We first aimed to confirm whether players who are familiar with loot boxes in the game *Overwatch* systematically categorize the value of the items in loot boxes based on the rarity of the items within the box. Although intuitively it would seem that this should be the case it is important to demonstrate this relation since other factors could potentially be at play—some loot boxes may be valued if they contain common items that nonetheless have a personal relevance to a particular player based on the character they typically play. To demonstrate that there was indeed a systematic relation between rarity and perceived value, we assessed the correlation between the net worth of the in-game items contained in a box as calculated using in-game currency with participants’ subjective ratings of that loot box’s value. Since the in-game currency measure is determined by the rarity of the items, if players do systematically value loot boxes containing rarer items more than loot boxes with less rare items, then we expect the subjective ratings of loot box value to track with its assigned objective value. The second aim of Experiment 1 was to determine whether loot boxes of greater objective and subjective value would yield higher ratings for arousal, positive valence, and urge to open another box. Overall we expected players to respond to loot boxes of greater objective and subjective value to be more arousing, more positively valenced, and importantly, more inducing of urge to open another loot box.

## Methods

### Participants

We recruited a total of 57 participants from two pools of students at the University of Waterloo. Twenty-eight participants were recruited from a pool of students voluntarily participating in psychology studies for credit. The remaining 29 student participants were recruited from poster advertisements across the University of Waterloo campus. In order to participate, students were required to have played the game *Overwatch* at least once in the past 4 weeks, as well as opened a loot box within *Overwatch* at least once in the past 4 weeks. Participants were compensated $5 for their time.

We excluded 10 participants due to incomplete data or failed attention checks. This left us with a final sample of 47 participants.

### Apparatus

#### Loot Box Stimuli

**Fig. 1:**
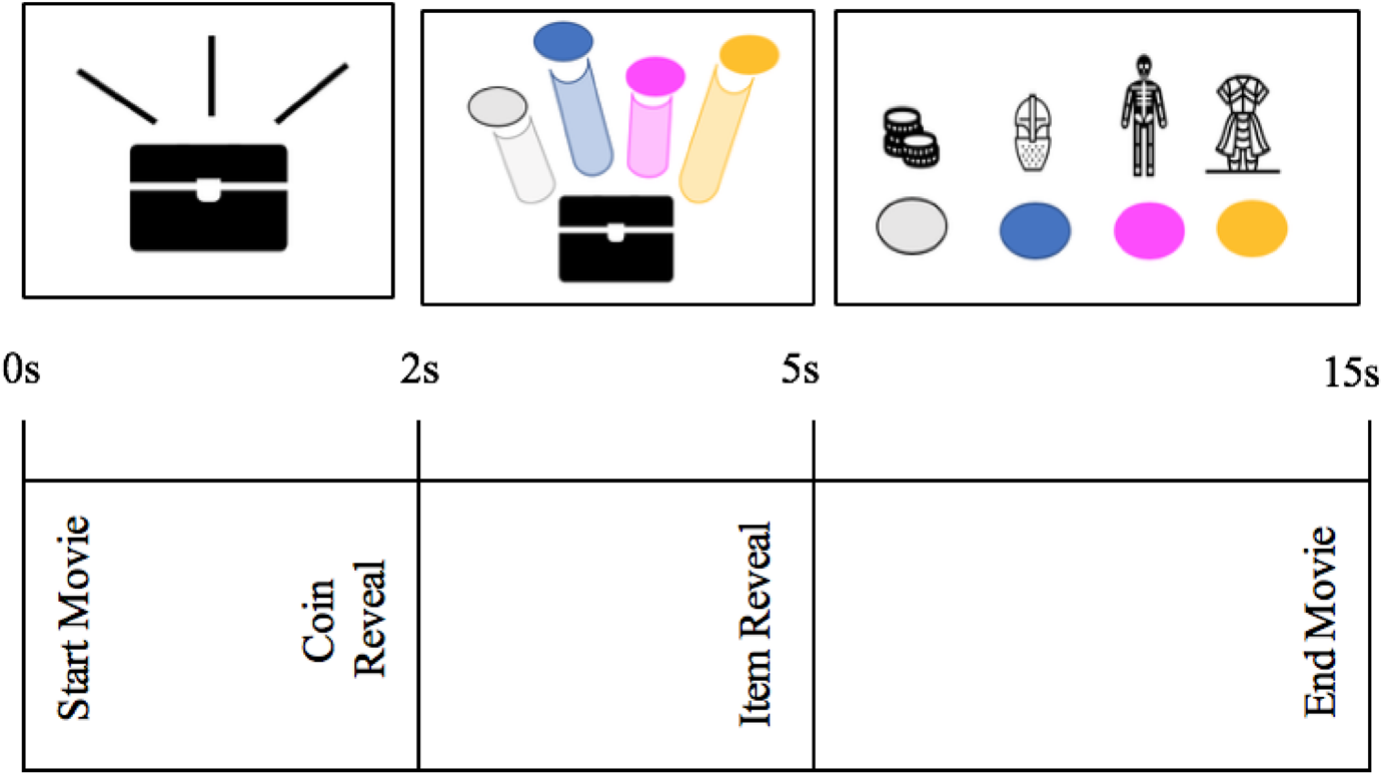
Depiction of loot box video event. Loot box opening begins at 0 s, the coin reveal begins at 2 s and the full item reveal at 5 s. Colours associated with each item are visible to players during both the coin reveal and item reveal periods

Participants viewed 49 videos of actual *Overwatch* loot box openings. Each video was a total of 15 s in length. In the first 2 s of each video, the loot box would appear to shake, at about 2 s the loot box would release four coloured coins representing each item into the air. The full reveal of the four loot box items occurred 5 s into the video (see Fig. 1). The video trial presentations appeared in randomized order for all participants.

To calculate the objective value of each loot box, we used the objective cumulative worth of all the items based on their individual credit worth in the game. Individual items belong to one of four possible classes based on its rarity in the game, signified by the colour of the coin shown during the Coin Reveal. The coins then become the platform beneath each item once they are revealed during the Item Reveal phase. The four classes in order of increasing rarity are as follows: common, rare, epic and legendary. However, by the game’s standards, each loot box guarantees at least one ‘rare’ tier item, allowing for the classification of boxes into three categories (Carpenter 2017). These ‘rare’, ‘epic’ and ‘legendary’ demarcations are used by the game designers and are familiar to avid players. Thus, we used the same classification system to categorize our stimulus set. Each class corresponds to a particular value of in-game credits. Furthermore, each class is associated with a specific colour highlighting the in-game value of the received items to players (see Table 1). For instance, the “rare” tier consisted of boxes containing at least one “blue” item and no epic (i.e., magenta) or legendary (i.e., gold) items, conferring a value range of 150 to 225 credits (a full list of the objective loot box values in our stimulus set can be found in supplementary materials). The finalized stimulus set consisted of 29 rare boxes, 15 epic boxes and 5 legendary boxes. These frequencies correspond with the actual probabilities of loot boxes of these values in the game, given that the stimuli were derived from a player’s single loot box opening session.

**Table 1:** Loot box tiers and value ranges

Tier	Criteria	n	Value range (net worth of all items in box)
Rare	Box contains at least one “Blue” item.	29	150–225 credits
Epic	Box contains at least one “Magenta” item.	15	325–500 credits
Legendary	Box contains at least one “Gold” item.	5	1075–1325 credits

### Materials

#### Subjective Ratings of Arousal and Valence

**Fig. 2:**
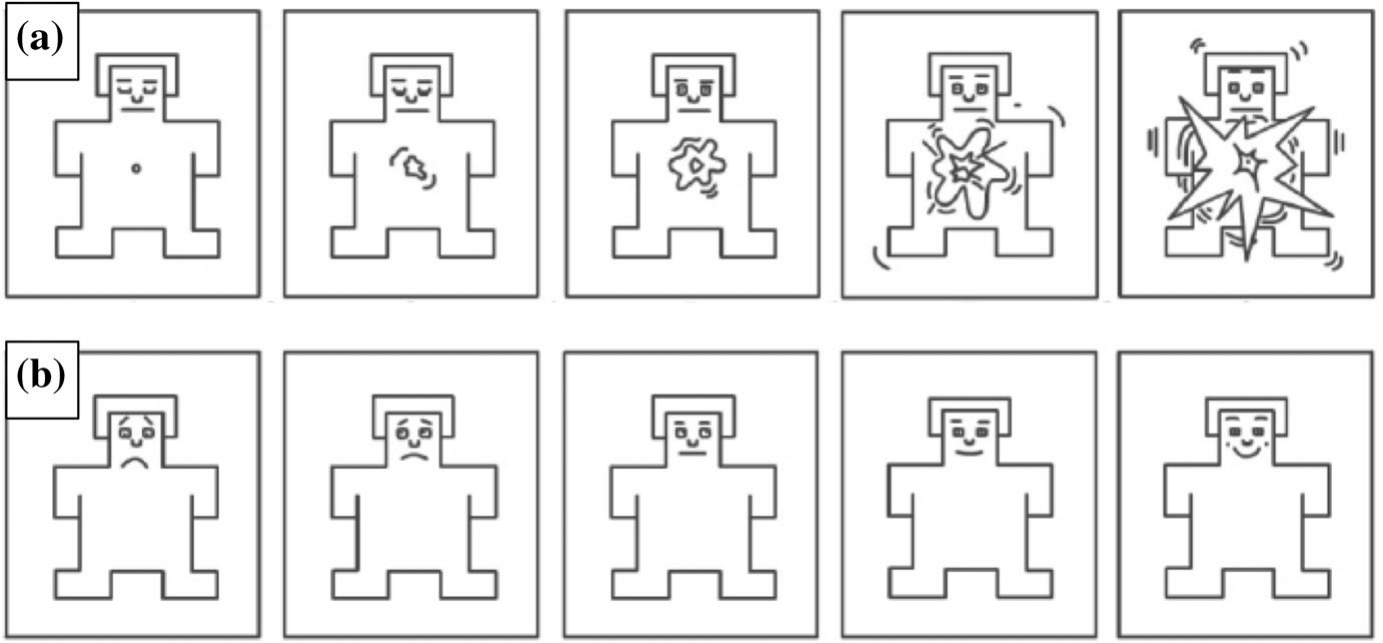
**a** Self-assessment manikin used to indicate subjective feelings of arousal. **b** Self-assessment manikin used to indicate subjective feelings of affect

Subjective ratings of arousal and valence were measured using Self-Assessment Manikins (SAM; Bradley and Lang 1994). Ratings of arousal and valence were measured for each loot box event. Participants indicated their current emotional state from a range of five manikins, which depicted an image representing varying levels of arousal and positive/negative valence (see Fig. 2).

#### Subjective Ratings of Urge to Open Another Loot Box

Urge to open another loot box was measured using a 100-point line scale, with 0 representing no urge and 100 representing high urge. Participants rated their urge after each loot box event via a mouse click along the line in response to an item that read ‘Using the scale below (0–100), please rate your level of urge to open another loot box’.

#### Loot Box Subjective Value

In order to gauge subjective value, participants were asked to indicate the number of in-game credits they would be willing to spend on the loot box using a number line. Loot box credits ranged from 0 credits (no value) to 4000 credits (high value). This 4000 point scale was used because this was the maximum amount obtainable, given that each box contained 4 items, with each item being worth up to 1000 credits. Participants were also asked to indicate their subjective worth on a scale from 1 (no worth) to 16 (high worth). Due to the issues of multicollinearity of this measure with subjective value, we have omitted the measure of subjective worth from further analysis.

### Procedure

This experiment was conducted using the online survey platform Qualtrics. Participants recruited from the university’s online research pool were redirected to the experiment, and immediately granted half a credit toward a course of their choosing upon completion. Participants recruited from the poster ads were asked to email researchers for a link to the survey.

Upon completing the online consent form, participants immediately began the experiment phase. Participants were presented with the randomized set of 49 loot box opening videos, each of them followed by the subjective survey battery. They were required to watch each video from start to finish before proceeding to the subjective surveys. Each subjective survey set contained a photographic depiction of the loot box outcome from the most recently opened loot box, as well as questions regarding their level of arousal, their subjective valence, and urge to open another box. Participants also indicated how much they were willing to spend for the items in each box. Participants completed the same survey set for all 49 videos, and were then debriefed.

## Experiment 1 Results and Discussion

### Data Reduction and Analysis Strategy

Out of the 57 participants recruited, only 47 had valid data for all trials and had passed the attention check. Outliers were removed using the Van Selst and Jolicoeur (1994) trimming procedure.

Subjective responses were analyzed by comparing the different tiers of the loot boxes to which participants were exposed. For all measures, loot outcomes that fell into their respective tiers were trimmed for outliers and then averaged. Using ‘arousal’ as an example, an outlier free average was calculated for the 29 ‘rare’ loot boxes, another outlier free average was calculated for the ‘epic’ loot boxes and a third outlier free average was calculated for the ‘legendary’ loot boxes. These averages from each participant were used as input data for a repeated measures analyses of variance (ANOVA) with tier (rare, epic, legendary) as the repeated factor. Any significant main effects were analyzed using Fisher’s Least Significant Difference (LSD) post hoc comparisons. Greenhouse–Geisser corrections were used when violations of sphericity occurred.

### Objective and Subjective Loot Box Values—Validity Check

The 49 loot boxes ranged in objective value from 150 game credits to 1325 game credits. Some loot boxes had the same objective value. For instance, there are 19 loot boxes objectively valued at 150 credits, 2 loot boxes worth 175 credits and 7 loot boxes worth 200 credits. In total there were 14 unique values of loot boxes (150, 175, 200, 225, 325, 350, 375, 400, 425, 450, 500, 1075, 1125, and 1325). To determine whether these objective loot box values correlated with participants’ subjective ratings of value, we tabulated the subjective ratings of value for the 14 aforementioned loot box values (e.g., an average subjective value was calculated for the 19 loot boxes objectively valued at 150 credits and used for this data point). Participants subjective ratings were then averaged culminating in 14 average ratings for each objective loot box value.

There was a strong, positive association between the objective and subjective values of the loot boxes measured in credits, *r*(13) = .962, *p* ≤ .001. This correlation demonstrates that these frequent players would pay more in game currency for loots that are objectively worth more according to the net worth of the loot boxes. Moreover, Pearson’s r correlations also showed that the subjective ratings are strongly and positively correlated with arousal (*r*(13) = .959, *p* ≤ .001) positive valence (*r*(13) = .938, *p* ≤ .001), and urge (*r*(13) = .905, *p* ≤ .001) to continue opening loot boxes. Objective ratings were also positively correlated with arousal, valence and urge. This pattern of results shows that more valuable loot boxes are deemed more arousing, positively valenced and inducing of the urge to open loot boxes.

**Fig. 3:**
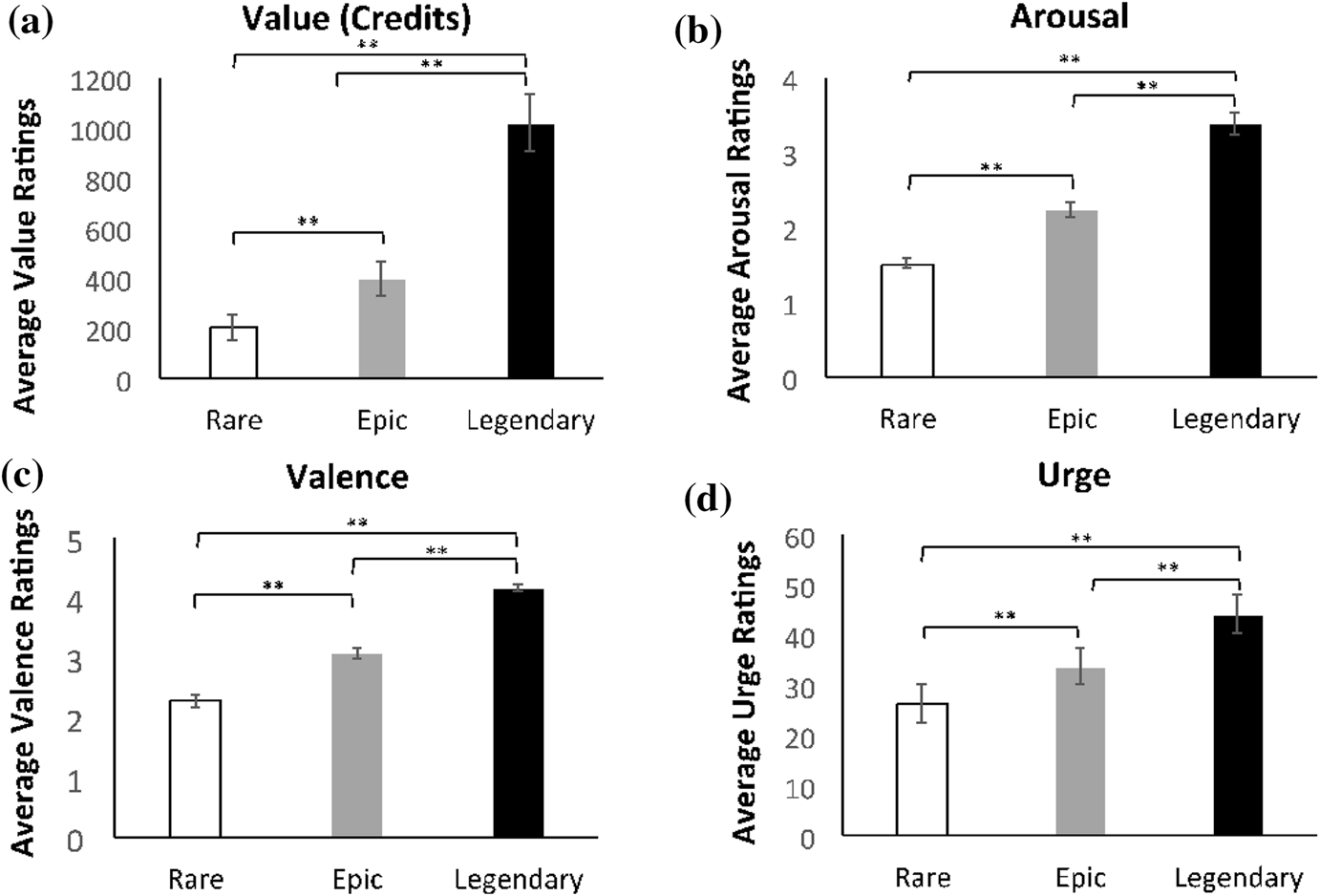
Experiment 1 subjective responses for loot boxes across different reward tiers (see Table 1 for tier value specifications). **a** Average subjective value in credits, **b** average subjective arousal ratings, **c** average ratings of valence, **d** average ratings of urge. Error bars ± 1 SE. * *p* ≤ .05; ** *p* ≤ .001

There was a consistent pattern of ratings for reward value when observing average ratings of loots across the reward tiers. Specifically, there was a main effect of credit value across the three different reward tiers, *F*(1.17, 54.82) = 59.53, *p* ≤ .001, *η*_*p*_^2^ = .564. As predicted, legendary loots (*M* = 987.15, *SD* = 807.85) were deemed the most valuable compared to loots that fell into the epic (*M* = 347.06, *SD* = 509.58; *p* ≤ .001) and rare categories (*M* = 188.56, *SD* = 360.89; *p* ≤ .001). Moreover, rare tier loots were the least valued and rated lower in comparison to epic loots (*p* ≤ .001). Here we provide evidence that players are indeed determining the value of the loots based on the rarity of the items depicted by the game, as opposed to other idiosyncratic factors such as how well an item would suit a particular a given player’s personal avatar (see Fig. 3).

### Subjective Ratings of Arousal, Valence and Urge

Figure 3 also displays the average ratings for arousal, valence, and urge across the three reward tiers of loot boxes.

In terms of arousal, a repeated measures ANOVA with a Greenhouse–Geisser correction revealed that there was a significant main effect of arousal across the three reward tiers, *F*(1.48, 68.19) = 125.28, *p* ≤ .001, *η*_*p*_^2^ = .731. As expected, Fisher’s LSD comparisons demonstrated that loot boxes containing at least one legendary item (*M* = 3.39, *SD* = 1.04) showed the greatest arousal scores compared to epic loot boxes (*M* = 2.21, *SD* = .753; *p* ≤ .001) and rare loot boxes (*M* = 1.46, *SD* = .487; *p* ≤ .001). Additionally, epic loot boxes were deemed more arousing to players compared to rare loot boxes (*p* ≤ .001).

For valence, a repeated measures ANOVA with a Greenhouse–Geisser correction revealed a significant main effect of valence across the different reward tiers, *F*(1.49, 68.85) = 166.87, *p* ≤ .001, *η*_*p*_^2^ = .784. Fisher’s LSD comparisons indicated that legendary loot boxes (*M* = 4.21*, SD* = .585) were rated the most positively valenced compared to epic (*M* = 3.09, *SD* = .649; *p* ≤ .001) and rare loot boxes (*M* = 2.26, *SD* = .802; *p* ≤ .001). Epic loot boxes, by contrast were more positively valenced than rare loot boxes (*p* ≤ .001).

Finally, a repeated measures ANOVA with a Greenhouse–Geisser correction revealed a significant main effect of urge across the reward tiers, *F*(1.22, 56.50) = 23.89, *p* ≤ .001, *ƞ*_*p*_^2^ = .349. As expected, Fisher’s LSD indicated that urge ratings were highest after experiencing a legendary loot box (*M* = 44.24, *SD* = 27.06) compared to an epic (*M* = 32.77, *SD* = 25.34; *p* ≤ .001) and rare loot box (*M* = 25.39, *SD* = 27.17; *p* ≤ .001). Epic loot boxes were more urge inducing compared to a rare item (*p* ≤ .001). The subjective ratings of value, arousal, valence and urge are indeed titrated to the size of the reward (or based on the tier to which they belong, based on their objective value).

In summary, in Experiment 1 we distributed an online survey containing 49 loot box videos and examined *Overwatch* players’ subjective ratings of value, arousal, valence and urge to open another box for each video. Players subjectively valued most those loot boxes with the highest objective worth (e.g., those that contained at least one of the most uncommon ‘legendary’ items) compared to loots that were objectively worth less (e.g., those containing more common items falling into the ‘rare’ and ‘epic’ tiers) (Fig. 3). Moreover, players also gave larger ratings of arousal, valence and urge as the reward value of the loot box increased (see Fig. 3).

## Overview of Experiment 2

We showed in Experiment 1 that players systematically categorized valuable and non-valuable loots based on increasing rarity, and hence increasing objective value, of the items in a loot box. We also showed that loots of increasing rarity were associated with greater arousal, were more positively valenced and more urge inducing. In Experiment 2, we sought to replicate this pattern of subjective responses of value, arousal, valence and urge. Since loots containing common items (those in the ‘rare’ tier) were the most negatively valenced, a measure of disappointment was introduced as a more nuanced assessment of negative valence. We therefore expect that more common items acquired in loot boxes should produce higher ratings of disappointment (van Dijk 1999).

Additionally, we sought to determine whether these subjective ratings converge with prominent measures of hedonic reward and arousal. Specifically, we examined whether the most uncommon items were hedonically the most rewarding as indexed by post-reinforcement pauses (PRPs). We predicted that loots containing exceedingly uncommon items (e.g., items in the legendary reward tier) would be more rewarding, hence, producing longer PRPs. We also examined whether video game players found these more valuable loots to be more arousing events as indexed by skin conductance responses (SCRs) and force responses. If so, the opening of valuable boxes should trigger larger SCRs, and such reward related arousal should manifest in harder mouse button presses as they press to continue the experiment and view more boxes. Specifically then both SCRs and force on the mouse button should rise with the rarity of the loot box just opened. Arousal should not only follow the opening of loot boxes, but should also heighten in the moments just before a loot box is opened. During this anticipation phase, when players might or might not see uncommon (and hence) valuable items, we would expect a rise in anticipatory arousal quantified by increases in skin conductance levels (SCLs).

## Methods

### Participants

A total of 46 avid Overwatch players from two participant pools were recruited to participate in the experiment. Of those 46, 37 participants were recruited through the University of Waterloo’s SONA system for partial course credit. The remaining 9 participants were recruited from posters advertising the experiment placed around the University of Waterloo campus and received $10 as financial remuneration for their time. Eligibility requirements were identical to Experiment 1.

### Apparatus

#### Loot Box Stimuli

We employed the same battery (n = 49) of video stimuli used in Experiment 1, plus three additional “practice” loot box videos used for familiarizing participants to the experimental protocol (the latter were not analyzed).

#### Post-Reinforcement Pauses

Participants were not required to watch each video in its entirety. Rather, they could click on a modified mouse to advance to the next stage of the experiment (the answering of subjective questions about the video they had just seen). Post-Reinforcement Pauses (PRPs) were based on how long players waited before clicking this modified mouse. Concretely, PRPs were measured by time in seconds between the reveal of the coloured coins and when they clicked on the modified mouse.

#### Skin Conductance Responses

Skin Conductance Responses (SCRs) were recorded via the use of two electrode plates (MLT118F GSR Finger Electrodes) attached to the index and middle finger of the participant’s non-dominant hand. The electrodes were fed into a Powerlab (model 4/30), which amplified the signal and converted the analog signal to a digital recording of participants’ physiological responses.

#### Force

Force was quantified as the amount of pressure (mv) imparted on the modified mouse when the participant made the press response to initiate the subjective surveys following the loot box video.

### Materials

#### Subjective Value, Arousal, Valence and Urge to Open Another Box

Items measuring subjective value, arousal, valence and urge were identical to what was used in Experiment 1.

#### Subjective Ratings of Disappointment

Disappointment was measured using a 100-point line scale, with 0 representing no disappointment and 100 representing high disappointment. Participants rated their disappointment after each loot box event via a mouse click along the line.

### Design

The experiment utilized a within-subjects design where following viewing 3 practice loot box videos and answering subjective questions to these videos participants were presented with 49 experimental loot box trials. Each participant viewed all 49 boxes, which consisted of outcomes ranging in value from 150 credits to 1325 credits.

### Procedure

After informed consent was provided, participants completed a demographic questionnaire using Qualtrics software on a PC computer for reasons peripheral to the current research. Upon completion, participants were instructed to face a separate Macintosh computer where the loot box trials took place. The researcher attached two electrodes to the middle and index fingertips of the participant’s non-dominant hand. The researcher instructed the participant to keep the hand attached to the electrodes as still as possible throughout this phase of the experiment.

Participants were then instructed to view each loot box video and to click the modified mouse when they were ready to move on to the subjective measures pertaining to the video they had just seen. Upon completion of these subjective questions, a new loot box video appeared. Participants were told that the first three loot boxes would be practice and as such could ask questions for clarification before moving onto the experimental trials. Once participants had viewed and completed the questionnaires for all 49 experimental trials, the experiment concluded.

## Experiment 2 Results and Discussion

Out of the 46 participants recruited, only 40 had valid data for all physiological and subjective measures. One participant was excluded for clicking the mouse (i.e., advancing to the subjective questions) prior to the reveal of the coins for 8 or more videos (15% of trials). Five participants were excluded for pressing the modified mouse button so softly that it failed to be recorded.

### Data Reduction and Analysis Strategy

Both physiological and subjective measures were subjected to outlier analyses. Outliers were determined using the Van Selst and Jolicoeur (1994) trimming procedure which removes the biases in outlier attribution due to different numbers of observations across conditions. This technique was necessary as there were a greater number of loot boxes with more common items than loot boxes with rare items in this experiment. Moreover, for non-excluded participants, if there were any trials where participants pressed the modified mouse to initiate the subjective surveys prior to the reveal of the coins, these trials were excluded from all further analyses since participants would not have viewed any information relevant to the value and contents of the loot box.

Reactions to the items within the loot box were measured using SCR amplitudes (Dawson et al. 2007). Recall that the coin reveal occurred at the 2 s mark of the video. We defined a 6 s window following the coin reveal (from the 3 s to 9 s marks in the video) in which changes in eccrine gland activity are attributable to viewing the coins or the appearance of the items themselves at the 5 s mark. We then subtracted the value at the beginning of this window from the maximum SCR within this window. The resulting value was the SCR amplitude related to the items in the loot box. Following Dawson et al. (2007), in calculating SCR amplitudes we considered as valid responses only those responses that were accompanied by an increase in skin conductance levels greater than .05 microsiemens.

Subjective responses were analyzed in an identical manner to Experiment 1. SCRs and PRPs were also analyzed using a similar strategy as the subjective responses. That is, for all subjective, physiological and behavioural measures, loot outcomes that fell into their respective tiers (rare, epic and legendary) were trimmed for outliers and then an average score was calculated for boxes within each tier. Repeated measures ANOVAs with Fisher’s LSD post hoc comparisons were conducted for each measure. Greenhouse–Geisser corrections were again used in cases of sphericity violations.

**Fig. 4:**
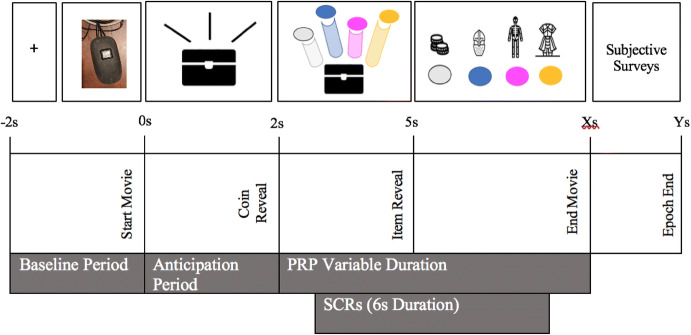
Depiction of events relevant to the particular measures. For anticipatory arousal, a baseline period consisted of 2 s prior to the onset of a video stimulus, and the anticipatory period occurring from seconds 1 to 2. PRPs were sampled within a variable time window between the onset of the items (2 s) and the end of the opening (7 s maximum). Six second SCR epochs were sampled 1 s after the “coin reveal”

Increases in skin conductance levels due to the anticipation of loot box openings were based on a 4 s window comprised of a baseline epoch (2 s window that occurred before the presentation of a loot box) and an anticipatory epoch (2 s window which depicted the loot box shaking prior to the reveal of its contents). Changes in SCLs for the baseline and the anticipation period were measured using SCL slopes and were directly compared for each trial using dependent *t* tests (Fig. 4).

### Objective and Subjective Loot Box Values—Validity Check

As in Experiment 1, a Pearson correlation between the 14 objective loot values and 14 final averaged subjective values for these boxes were strongly positively correlated (*r* (13) = .95, *p* ≤ .001).

**Fig. 5:**
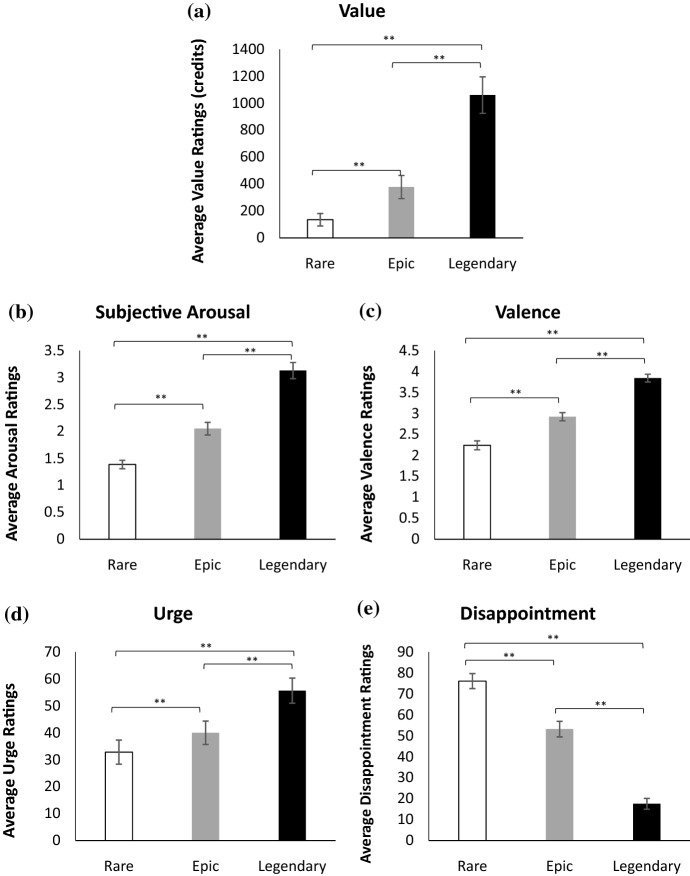
Results from subjective measures. **a** Average subjective ratings of loot box value (in credits) for rare, epic and legendary tier loots. **b** Average ratings of subjective arousal for Rare, Epic and Legendary tier loot boxes. **c** Average ratings of subjective affect for Rare, Epic and Legendary tier loot boxes. **d** Average ratings of subjective urge to open additional loot boxes for Rare, Epic and Legendary tier loot boxes. **e** Average ratings of subjective disappointment for Rare, Epic and Legendary tier loot boxes. *Error bars* are ± 1 SE. * *p* ≤ .05; ** *p* ≤ .001

Results for the scales assessing subjective value illustrated the expected pattern of increasing value from rare to legendary tiers. For subjective ratings of value (in credits), a repeated measures ANOVA with a Greenhouse–Geisser correction illustrated a significant main effect of condition, *F*(1.243, 48.471) = 52.00, *p* ≤ .001, *η*_*p*_^2^ = .57. Fisher’s LSD comparisons revealed that participants rated themselves as willing to spend the greatest amount of in-game credits for legendary tier loots (*M* = 1059.47, *SD* = 857.30) and the least amount for rare tier loots (*M* = 135.35, *SD* = 292.81). Ratings of value for the legendary tier were significantly greater than the epic tier (*M* = 377.83, *SD* = 542.83) (*p* ≤ .001) and the rare tier (*p* ≤ .001). Additionally, ratings of value were significantly higher for the epic tier in comparison to the rare tier (*p* ≤ .001). See Fig. 5 for mean subjective value ratings.

### Subjective Measures

Average ratings of subjective measures are shown in Fig. 5. A repeated measures ANOVA with a Greenhouse–Geisser correction was conducted for subjective ratings of arousal, showing a significant main effect of condition, *F*(1.371, 53.486) = 141.21, *p* ≤ .001, *η*_*p*_^2^ = .78. As expected, Fisher’s LSD comparisons revealed that subjective ratings of arousal were greatest for legendary tier loots (*M* = 3.13, *SD* = .95) and lowest for rare tier loots (*M* = 1.39, *SD* = .49). Ratings of arousal for legendary tier loots were significantly greater than epic tier loots (*M* = 2.05, *SD* = .74) (*p* ≤ .001) and significantly greater than rare tier loots (*p* ≤ .001). Further, arousal ratings for epic tier loots were significantly greater than rare tier loots (*p* ≤ .001).

A repeated measures ANOVA with a Greenhouse–Geisser correction conducted on subjective measures of valence illustrated the same pattern of results. There was a main effect of condition, *F*(1.506, 58.722) = 101.68, *p* ≤ .001, *η*_*p*_^2^ = .72. Fisher’s LSD comparisons also revealed that positive valence was greatest for legendary tier loots (*M* = 3.84, *SD* = .58) and lowest for rare tier loots (*M* = 2.24, *SD* = .67). legendary tier loots were associated with significantly greater ratings of positive valence than epic tier loots (*M* = 2.92, *SD* = .61) (*p* ≤ .001) and rare tier loots (*p* ≤ .001). Positive valence ratings for epic tier loots were also significantly greater than rare tier loots (*p* ≤ .001).

Subjective ratings of urge to open additional loot boxes also corresponded to this pattern of an increase in scores across the three tiers. A repeated measures ANOVA with a Greenhouse–Geisser correction illustrated a main effect of condition, *F*(1.317, 51.379) = 24.02, *p* ≤ .001, *η*_*p*_^2^ = .38. Fisher’s LSD post hoc comparisons showed greatest urge with the legendary tier (*M* = 55.63, *SD* = 29.46) and least urge with the rare tier (*M* = 32.84, *SD* = 28.30). Urge ratings for legendary tier loots were significantly greater than urge ratings for epic tier loots (*M* = 40.00, *SD* = 27.42) (*p* ≤ .001) and rare tier loots (*p* ≤ .001). Moreover, urge ratings for epic tier loots were significantly greater than urge ratings for rare tier loots (*p* ≤ .001).

As predicted, results from ratings of disappointment revealed a monotonic decrease in average scores from rare to legendary tiers. A repeated measures ANOVA with a Greenhouse–Geisser correction revealed a significant main effect of condition, *F*(1.610, 62.802) = 149.77, *p* ≤ .001, *η*_*p*_^2^ = .79. Fisher’s LSD comparisons showed the most disappointment for rare tier loots (*M* = 76.07, *SD* = 22.69) and the least disappointment for the legendary tier (*M* = 17.55, *SD* = 16.27). The legendary tier had significantly lower ratings of disappointment than the epic tier (*M* = 53.18, *SD* = 23.43) (*p* ≤ .001) and the rare tier (*p* ≤ .001). Ratings of disappointment were also significantly lower for epic tier loots than rare tier loots (*p* ≤ .001).

### Physiological and Behavioural Reactions to Loot boxes

**Fig. 6:**
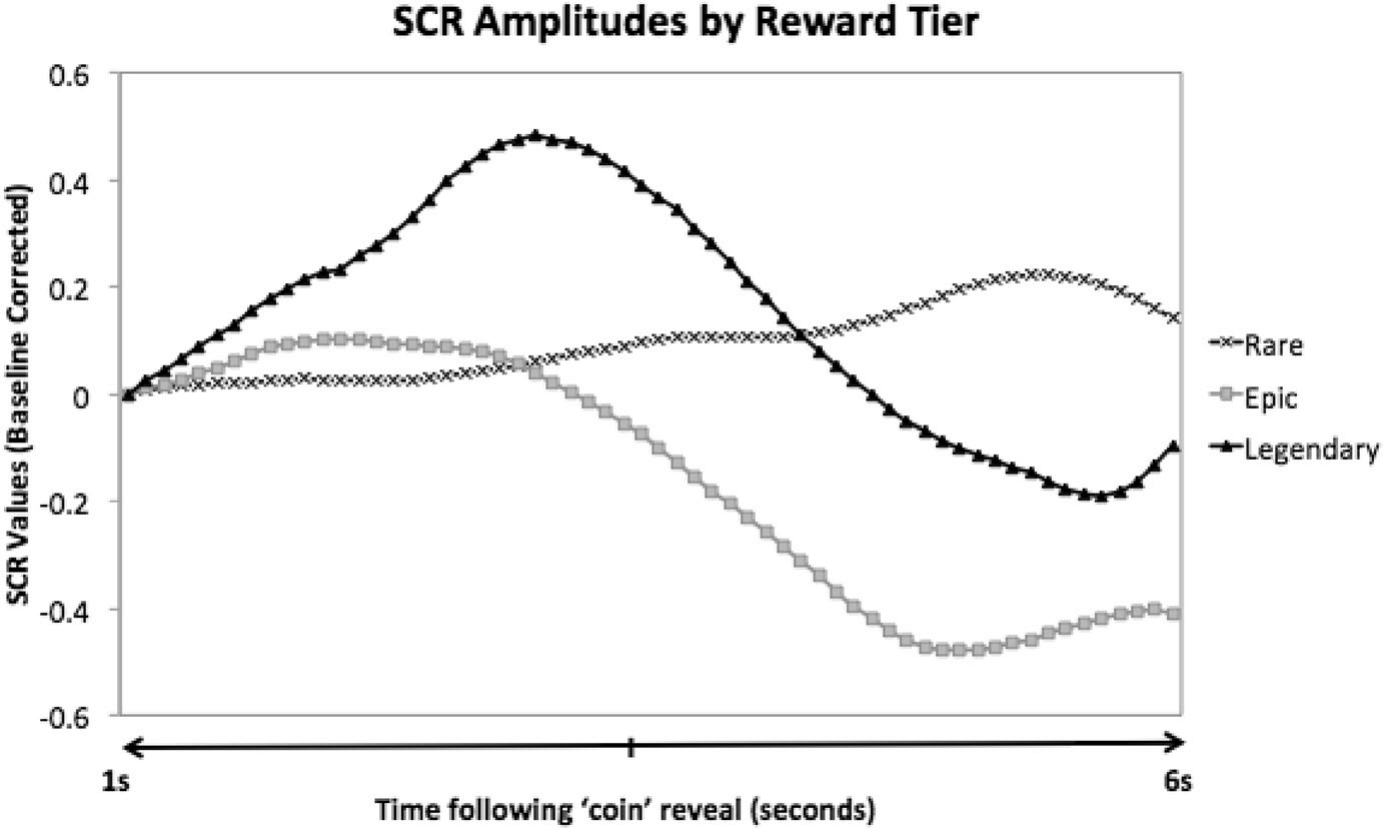
Raw SCR values over 6 s following the ‘coin reveal’ of a loot box opening for a representative participant (determined by the median response average for legendary loots). The raw values depict the median participants’ average amplitudes after viewing a legendary, epic, and rare tier loot box respectively. For all trials, participants SCLs were forced to zero via subtraction at the beginning of the SCR window—thus the figure shows changes in SCL over the 6 s window

A repeated measures ANOVA with a Greenhouse–Geisser correction revealed a significant main effect of condition for SCR amplitudes, *F*(1.232, 46.816) = 11.39, *p* ≤ .001, *η*_*p*_^2^ = .23. Further, Fisher’s LSD comparisons revealed no significant difference between the rare tier loots (*M* = .58, *SD* = .28) and epic tier loots (*M* = .54, *SD* = .29) (*p* = .08). However, SCR amplitudes in response to legendary tier loots (*M* = .77, *SD* = .49) were significantly greater than those for rare tier loots (*p* ≤ .05) and epic tier loots (*p* ≤ .05). See Fig. 6 for a representative example of the sizeable SCR amplitude for legendary loots compared to the epic and rare loots.

Similarly, there was a main effect of condition for the force with which participants pressed the modified mouse, *F*(1.433, 55.903) = 4.53, *p* ≤ .05, *η*_*p*_^2^ = .10 (with a Greenhouse–Geisser Correction). Fisher’s LSD comparisons found no significant difference in force between the rare tier loots (*M* = .13, *SD* = .03) and epic tier loots (*M* = .14, *SD* = .03) (*p* = .43). Similar to the SCR amplitudes, significantly greater force was found for legendary tier loots (*M* = .14, *SD* = .04) versus rare tier loots (*p* ≤ .05) and epic tier loots (*p* ≤ .05).

**Fig. 7:**
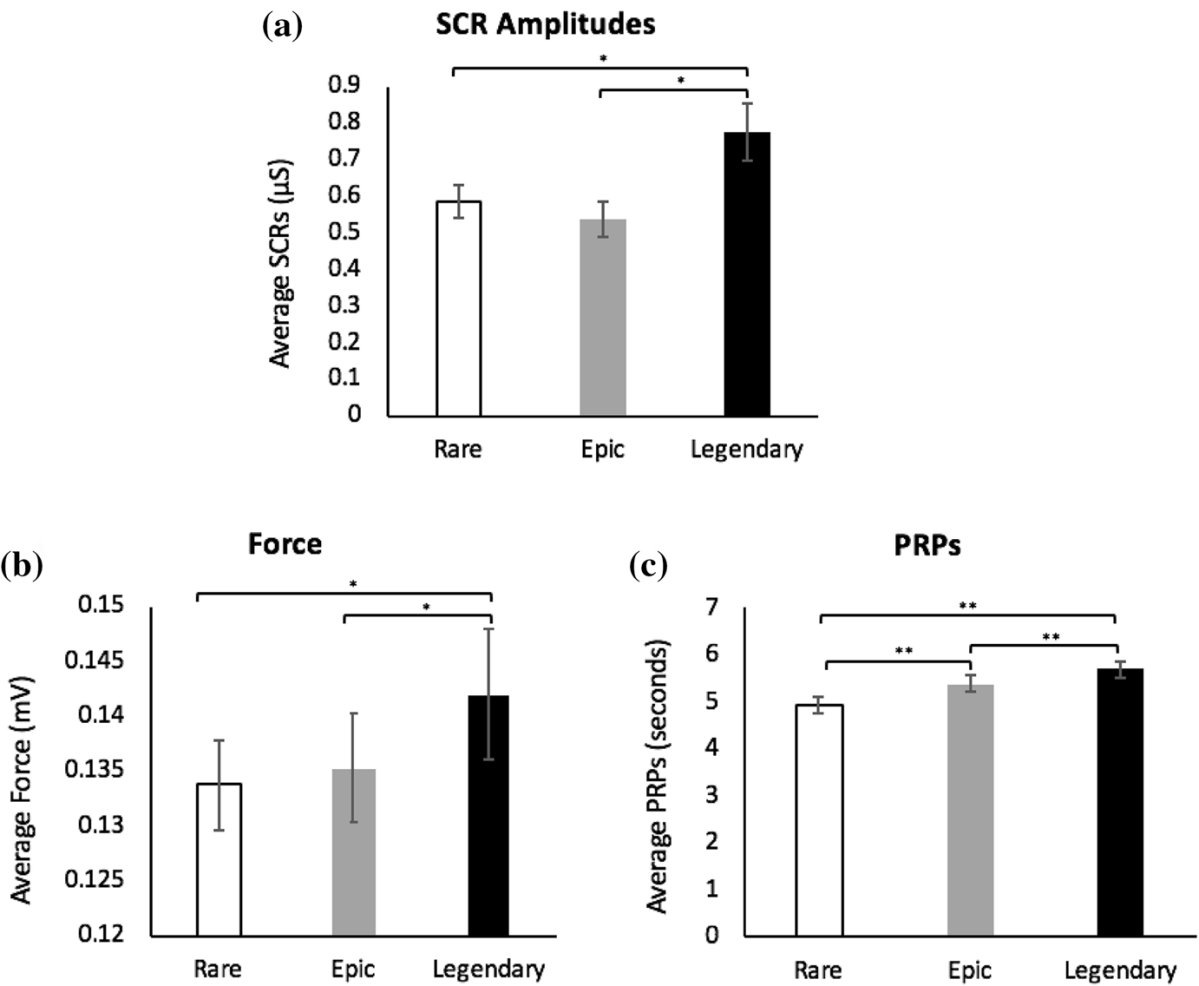
Results from physiological and behavioural measures. **a** Average participant SCR amplitudes for Rare, Epic and Legendary tier loot boxes. **b** Average force exerted on modified mouse to initiate the following loot box opening for Rare, Epic and Legendary tier loot boxes. **c** Average length of PRPs for Rare, Epic and Legendary tier loot boxes. *Error bars* are ± 1 SE. * p ≤ .05; ** p ≤ .001

Lastly, a repeated measures ANOVA with a Greenhouse–Geisser correction demonstrated a significant main effect of tier for PRPs, *F*(1.479, 57.667) = 21.16, *p* ≤ .001, *η*_*p*_^2^ = .35. Fisher’s LSD post hoc tests revealed that players had smaller PRPs for the rare tier (*M* = 4.91, *SD* = 1.09) in comparison to both epic (*M* = 5.40, *SD* = 1.05, *p* ≤ .001) and legendary tiers (*M* = 5.70, *SD* = 1.11, *p* ≤ .001). Epic and legendary also significantly differed in participant PRP lengths (*p* ≤ .001). See Fig. 7 for graphical illustrations of these physiological and behavioural measures.

### Anticipatory Arousal

**Fig. 8:**
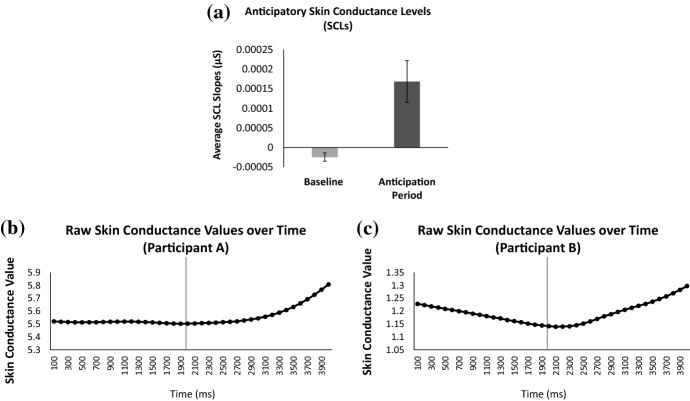
**a** Comparisons of participants’ physiological arousal between the baseline and anticipation period using average skin conductance slopes for all loot box events. **b**, **c** Raw skin conductance values of two median participants during the baseline and anticipation period. The vertical line denotes the transition from the baseline (before the loot box video starts) to the anticipatory period (after the loot box movie starts). *Error bars* are ± 1 SE. * p ≤ .05; ** p ≤ .001

A paired samples *t* test revealed significantly greater SCL slopes in the anticipatory period (*M* = .0002, *SD* = .0003) in comparison to the baseline period (*M* = -.00002, *SD* = .0001), *t*(39) = − 3.88, *p* ≤ .001 (see Fig. 8). The lower panels of Fig. 8 graphically depict the continuous changes in SCLs of two participants who fell at the median for SCL increases during the anticipation of the loot box reveal. This figure clearly shows a ramping up of of physiological arousal in anticipation of the loot box event (see Fig. 8).

## General Discussion

The current research aimed to characterize how loot box users respond to loot box rewards of varying value. We reasoned that if responses were similar to that reported for slots players reacting to varying sizes of monetary wins at the hedonic and motivational level, then it would indicate a need for loot boxes to be similarly regulated to prevent or reduce problematic usage. Participants were exposed to a series of video stimuli depicting loot box openings from the game *Overwatch*, with loots ranging in value based on the game’s reward tier hierarchy. For each opening, we gauged their subjective ratings of value, as well as subjective experiences of arousal, valence, and urge in Experiment 1. Experiment 2 employed the same subjective measures, while also measuring physiological and behavioural experiences of arousal and reward valence. In Experiment 1, we provide initial evidence that players systematically discriminate valuable from less valuable loots based on the rarity of the items, which corresponds with the game’s item value hierarchy (e.g., rare, epic and legendary tiers). Experiment 1 also showed that loots of greater rarity are subjectively more arousing, positively valenced and inducing of urge to open more boxes. Experiment 2 successfully replicated the results for these subjective measures, in addition to supplying converging evidence of the arousing, hedonically rewarding and motivating nature of these non-monetary rewards with PRPs, SCRs and force measures. Our data provide strong evidence for the allure of these non-monetary reward items, and the motivational impact such rewards have on players.

In contrasting players subjective value with the objective value of the loots, we showed that participants found loots containing items of greater rarity to be more valuable both at the group level (e.g., the means) and the individual level (e.g., correlations). Specifically, at the group level, the magnitude of the ratings of subjective value were titrated to the magnitude of the rarity across the three designated reward tiers. Similarly, correlations revealed that players’ ratings of subjective loot box value corresponded with the objective loot box values. This finding is an important confirmation of our assumption that loot boxes containing items of greater rarity would be more valuable to participants than more common items.

Similar to the indisputable, rewarding feeling of winning money, we show that obtaining in-game items within a loot box appear to activate the same reward responses as money in a slot machine (Dixon et al. 2013, 2015, 2018a, b). Our PRP results mimicked participants subjective “value” ratings for loots over the different reward tiers, such that there was a monotonic increase in pause length with increasing reward tier value. Specifically, loots in the legendary tier elicited longer pauses than the more common, lowest valued tier of loot boxes. Post-reinforcement pauses are seen as a direct measure of the hedonic pleasure associated with rewarding stimuli, and such PRP results mirror findings of greater PRPs following bigger wins in slots play (Dixon et al. 2013). Coupled with the subjective ratings of value, such findings are indicative of players’ awareness and sensitivity to the value of different loots, despite loot boxes not conferring any real-world monetary worth.

Our findings also suggest that items of the greatest rarity were the most subjectively and physiologically arousing, hedonically pleasing, and importantly the most inducing of urge to open another box. Specifically, subjective ratings of arousal, valence and urge all showed the same monotonic increase with increasing reward tier. Convergently, for disappointment, players showed a monotonic *decrease* with increasing reward tier (i.e., the most common loots were the most disappointing, the least common loots the least disappointing). Taken together, the fact that these subjective measures were yoked to the magnitude of the objective reward value suggest that the degree of positive excitement elicited for these events is related to the rarity of the loots in the game. Similarly, the legendary reward tier loots were associated with greater skin conductance responses and force (a complementary measure of positive arousal) compared to epic or rare loots. Thus, our subjective, physiological and behavioural indices of arousal converge to support the notion that the rarest loots (those falling in the legendary category) are the most rewarding, exciting and motivating events for players.

Unlike our subjective measures and PRP results, there was no differentiation in force magnitude nor skin conductance amplitude between loots corresponding to the epic and rare tiers. As force is typically quite sensitive to the reward magnitudes in slots (Dixon et al. 2015, 2018a, b), this lack of differentiation may be due to the smaller disparity between the value ranges of the rare and epic tier in comparison to the much larger disparity in value associated with legendary tier boxes. As can be seen in Table 1 the upper bound of the rare tier (225 credits) and the lower bound of the epic tier (325 credits) differ by only 100 credits whereas the upper bound of the epic tier (500 credits) and the lower bound of the legendary tier (1075 credits differs by 575 credits. Thus, it may be that ‘rare’ and ‘epic’ tiers defined by the game, are too similar to be differentiated by SCR and force measures. Importantly, both measures are convergently sensitive to the presentation of loot boxes containing the most uncommon items.

Even before seeing the items in the loot box, participants showed a marked increase in arousal in anticipation of the loot box opening. Previous research has illustrated increased skin conductance and activation of arousal-related brain regions during reward anticipation (Critchley et al. 2001). The finding that loot boxes elicit strong anticipatory arousal suggests that participants treat loot boxes as having the potential to confer reward. Such anticipatory arousal patterns are akin to player experience in slots play, such that there is a buildup in anticipation as the reels spin and sequentially settle (Dixon et al. 2011).

While there may be a build-up of anticipation in both slots and loot box openings, there are some subtle differences in the reveal of outcomes that may make the subjective feeling of anticipation distinct between the two games. For instance, slot machine reel symbols can be used as cues to index the proximity of a desired outcome as each reel sequentially settles. A classic example includes near-miss outcomes in slots—such outcomes are driven by cues that seemingly inform the player how close they are to their desired goal (e.g., a large win or a jackpot). In the case of loot boxes, game designers go to great lengths to illustrate general cues designed to increase arousal. In Overwatch, prior to displaying any contents the loot box is shown to tremble and shake—reminiscent and perhaps hoping to mirror one trembling with anticipation. To our knowledge there is no comparable feature in standard slot machine games.

Physiological arousal has been implicated in the maintenance of gambling behaviours across multiple modes of gambling, and our results for physiological arousal and urge dovetail with these previous findings from the gambling literature (Clark et al. 2012; Baudinet and Blaszczynski 2013; Stange et al. 2017a, b). The gradual ramping up of arousal and the fact that participants experienced additional increases in physiological arousal following the coin reveal (especially for higher value loots) corroborates players’ urge ratings and confirms the strong motivational force of these uncommon rewards. The urge to open more loot boxes following viewing of higher valued loot boxes may have implications for players’ behaviours regarding loot box use. For instance, there could be concern that increases in urge to open another loot box after receiving a valuable loot box during game play may invigorate players to access more loot boxes, either through continued gameplay (e.g., requiring an increased investment of time) or through purchasing (e.g., requiring increased monetary investment).

In summary, our research lends credence to previous commentaries and research suggesting that loot boxes are psychologically akin to gambling (Drummond and Sauer 2018; Brooks and Clark 2019). The current research is among the first to provide empirical evidence that the reveal of highly desirable items increases both arousal and more importantly urge to open more loot boxes, for the potential for problematic play in loot box games. Demonstrating such reward reactivity and urge for the rarest loots using loot box related cues is important for understanding how these gambling-like gaming features may result in problematic use, as they elicit responses that mirror those of foreknown addictive gambling forms. This is especially concerning when coupled with the structural similarities between loot boxes and slot machines, such as the use of a variable ratio reinforcement schedule. In variable ratio schedules, rewards are unpredictable and high valued (good) loots occur much less frequently than lower valued (bad) loots. This reward schedule framework has been associated with potentially maladaptive behaviours in gambling, and thus can potentially extend to loot boxes (Haw 2008). In most jurisdictions, loot boxes are very loosely regulated compared to legalized gambling activities. For one, gambling venues and websites are obligated to include help resources for gamblers who feel that their gambling behaviour is out of control. As the harms related to loot box use are becoming more salient, one direction for regulation could involve requiring games to feature similar safeguards. Another key regulatory discrepancy between games with loot boxes and gambling involves strictly enforced age regulations. Our results support the need for such age regulations for users under the legal gambling age.

### Limitations

This experiment is not without limitations. Firstly, we did not differentiate reactivity to these rewards among players who may potentially be at risk of excessive use of loot boxes. However, research has yet to solidify what constitutes a problematic loot box user, and thus, we are limited by the current research landscape. Secondly, in order to maximize experimental control, we used video stimuli rather than loot boxes that were obtained by the player. Future research should aim to replicate our findings in a more naturalistic setting using real loot boxes either won through game play, or purchased by the player. Given the added component of agency and ownership of rewards that are either earned by one’s own gameplay, or purchased with one’s own money, one might expect an amplification of the effects on the player that we have shown in this experiment which lacks such agency. Finally, since the presentation and valuation system of loot boxes is heterogeneous across games, future research should aim to reproduce our results using loot box stimuli from other games.

## Conclusion

In conclusion, our findings provide initial insight into the impact of loot box opening on player reward reactivity and motivation. Despite conferring no real-world value, loot boxes, especially those of greater rarity, are treated as rewarding and urge-inducing events. While the relationship between loot boxes, problem video gaming and problem gambling is still in need of further investigation, the consequences of such potential associations have profound implications for the future regulation of these and similar features in games.

## Electronic supplementary material

Below is the link to the electronic supplementary material.

Supplementary material 1 (DOCX 13 kb)
