# Supplementary material for: Rare Loot Box Rewards Trigger Larger Arousal and Reward Responses, and Greater Urge to Open More Loot Boxes
Source: J Gambl Stud. 2019 Nov 23;37(1):141–63. doi: 10.1007/s10899-019-09913-5 (PMC7882574; doi:10.1007/s10899-019-09913-5)
Supplement: Supplementary file 1 — Supplementary material 1 (DOCX 13 kb) [file 10899_2019_9913_MOESM1_ESM.docx]

Supplementary Materials

*Objective Loot Box Values*

| Loot Box | Item 1 Value (credits) | Item 2 Value (credits) | Item 3 Value (credits) | Item 4 Value (credits) | Total Box Value (credits) |
| --- | --- | --- | --- | --- | --- |
| 1 | 75 | 25 | 150 | 75 | 325 |
| 2 | 25 | 25 | 250 | 25 | 325 |
| 3 | 25 | 25 | 75 | 25 | 150 |
| 4 | 150 | 25 | 250 | 25 | 450 |
| 5 | 75 | 25 | 150 | 75 | 325 |
| 6 | 25 | 25 | 75 | 25 | 150 |
| 7 | 25 | 25 | 75 | 25 | 150 |
| 8 | 75 | 25 | 50 | 25 | 175 |
| 9 | 150 | 25 | 250 | 75 | 500 |
| 10 | 25 | 25 | 75 | 25 | 150 |
| 11 | 25 | 25 | 75 | 25 | 150 |
| 12 | 25 | 25 | 250 | 25 | 325 |
| 13 | 75 | 25 | 75 | 25 | 200 |
| 14 | 75 | 25 | 250 | 75 | 425 |
| 15 | 50 | 25 | 250 | 25 | 350 |
| 16 | 25 | 25 | 75 | 25 | 150 |
| 17 | 25 | 25 | 75 | 25 | 150 |
| 18 | 75 | 25 | 75 | 25 | 200 |
| 19 | 75 | 25 | 1000 | 25 | 1125 |
| 20 | 25 | 25 | 1000 | 25 | 1075 |
| 21 | 75 | 25 | 250 | 25 | 375 |
| 22 | 25 | 25 | 75 | 25 | 150 |
| 23 | 25 | 25 | 75 | 25 | 150 |
| 24 | 25 | 25 | 75 | 25 | 150 |
| 25 | 75 | 25 | 75 | 25 | 200 |
| 26 | 75 | 25 | 75 | 25 | 200 |
| 27 | 75 | 25 | 75 | 25 | 200 |
| 28 | 25 | 25 | 75 | 25 | 150 |
| 29 | 75 | 25 | 250 | 25 | 375 |
| 30 | 50 | 75 | 250 | 75 | 450 |
| 31 | 25 | 25 | 75 | 25 | 150 |
| 32 | 25 | 25 | 75 | 25 | 150 |
| 33 | 25 | 25 | 75 | 25 | 150 |
| 34 | 75 | 25 | 75 | 25 | 200 |
| 35 | 75 | 25 | 1000 | 25 | 1125 |
| 36 | 250 | 25 | 1000 | 50 | 1325 |
| 37 | 75 | 25 | 1000 | 25 | 1125 |
| 38 | 75 | 25 | 75 | 25 | 200 |
| 39 | 75 | 25 | 250 | 25 | 375 |
| 40 | 25 | 25 | 75 | 25 | 150 |
| 41 | 25 | 25 | 75 | 25 | 150 |
| 42 | 25 | 25 | 75 | 25 | 150 |
| 43 | 25 | 25 | 75 | 25 | 150 |
| 44 | 75 | 25 | 250 | 25 | 375 |
| 45 | 75 | 25 | 150 | 75 | 325 |
| 46 | 75 | 25 | 50 | 75 | 225 |
| 47 | 25 | 25 | 75 | 25 | 150 |
| 48 | 75 | 25 | 250 | 50 | 400 |
| 49 | 75 | 25 | 50 | 25 | 175 |
